# Mercury exposure and health challenges in Rapa Nui green turtles: urging conservation and long-term monitoring in the South Pacific

**DOI:** 10.1093/conphys/coaf019

**Published:** 2025-04-09

**Authors:** Rocío Álvarez-Varas, Eamy Ayala, Rocío Lagos, Irene Peña-Galindo, Victoria Palma-Rojas, Nels Hereveri, Nayade Campos, Gustavo Chiang, Carlos F Gaymer

**Affiliations:** Instituto de Biología, Facultad de Ciencias, Pontificia Universidad Católica de Valparaíso, Av. Universidad 330, Zip code 2373223, Valparaíso, Chile; Centro de Ecología y Manejo Sustentable de Islas Oceánicas (ESMOI), Departamento de Biología Marina, Facultad de Ciencias del Mar, Universidad Católica del Norte, Larrondo 1281, Zip code 781421, Coquimbo, Chile; Qarapara Tortugas Marinas Chile NGO, Las Flores Oriente 2725, Zip code 7940560, Santiago, Chile; Qarapara Tortugas Marinas Chile NGO, Las Flores Oriente 2725, Zip code 7940560, Santiago, Chile; Facultad de Ciencias, Universidad de Chile, Las Palmeras 3425, Zip code 7800003, Santiago, Chile; Laboratorio Clínico Veterinario SpVet, Arturo Prat 705, Zip code 9500037, Región Metropolitana, Santiago, Chile; Programa de Doctorado en Medicina de la Conservación, Facultad de Ciencias de la Vida, Universidad Andrés Bello, Av. República 440, Zip code 8370251, Santiago, Chile; Escuela de Medicina Veterinaria, Pontificia Universidad Católica de Chile, Av. Vicuña Mackenna 4860, Zip code 7820436, Santiago, Chile; Instituto de Biología, Facultad de Ciencias, Pontificia Universidad Católica de Valparaíso, Av. Universidad 330, Zip code 2373223, Valparaíso, Chile; Fishermen's Union Hanga Roa O'tai, Rapa Nui, Chile; TeMar Pacific Ocean Care NGO, Rapa Nui, Chile; Centro para la Resiliencia, Adaptación y Mitigación (CReAM), Universidad Mayor, Zip code 4801043 Temuco, Chile; Centro de Investigación para la Sustentabilidad, Facultad de Ciencias de la Vida, Universidad Andrés Bello, Av. República 440, Zip code 8370251, Santiago, Chile; Centro de Ecología y Manejo Sustentable de Islas Oceánicas (ESMOI), Departamento de Biología Marina, Facultad de Ciencias del Mar, Universidad Católica del Norte, Larrondo 1281, Zip code 781421, Coquimbo, Chile

**Keywords:** Chelonia mydas, conservation, Easter island, Hg, one health, pollution, public health

## Abstract

The endangered green sea turtle (*Chelonia mydas;* hereafter *C. mydas*) plays a crucial role in maintaining the balance of marine ecosystems. However, its populations are highly vulnerable to various threats, including marine pollution. Rapa Nui (Easter Island), an isolated location in the southeastern Pacific, provides vital foraging habitats for both morphotypes of Pacific *C. mydas* (black and yellow). In this study, we examined the demographic structure (morphotype, life stage, sex) and health status (based on blood analytes and mercury-Hg concentration) of *C. mydas* on Rapa Nui during 2018 and 2023. Turtles from various life stages and sexes were observed, with a predominance of yellow morphotype juveniles, likely recently recruited or emerging from brumation. Haematological analyses revealed low levels of several key analytes (e.g. cholesterol, calcium, phosphorus, total protein, globulins), suggesting poor nutritional status, potentially related to the brumation process, limited food availability or poor food quality in the region. Alterations in both red and white blood cell lines, including anaemia and lymphopenia, indicate ongoing inflammatory states and infections, consistent with clinical observations. Rapa Nui turtles exhibited some of the highest blood Hg concentrations globally. Abnormalities in blood profiles, along with correlations between various analytes and blood Hg concentrations, suggest altered immune function and probable renal and liver dysfunction, likely resulting from both natural and anthropogenic sources of this heavy metal. Additionally, a very high body condition index in turtles with carapace lesions suggests a negative impact from human food subsidies in local bays, particularly from high-trophic-level fish, which may also serve as a pathway for Hg accumulation, both for the turtle aggregation and the human population. Our findings underscore the urgent need for long-term mercury monitoring and turtle movement studies to identify pollution sources, inform effective conservation strategies for this endangered species, and address potential public health concerns on this remote Pacific island.

## Introduction

Rapa Nui (Easter Island) is a remote southeastern Pacific Island recognized as a centre of biodiversity and endemism (Roberts *et al.*, 2002) and declared a World Heritage Site by the United Nations Educational, Scientific and Cultural Organization in 1995 (Álvarez-Varas *et al.*, 2015). It is located in the centre of the South Pacific Gyre, being mostly affected by climatic drivers such as El Niño-Southern Oscillation (ENSO), the Southern Annular Mode and the Decadal and Inter-decadal Pacific Oscillation (Quilliam *et al.*, 2014).

Easter Island provides developmental and foraging habitats for four of five sea turtle species reported in the Eastern Pacific Region: loggerhead (*Caretta caretta*), leatherback (*Dermochelys coriace*a), hawksbill (*Eretmochelys imbricata*), and green sea turtle (*Chelonia mydas*). *Chelonia mydas* is an endangered species whose nesting rookeries are in tropical and subtropical areas, but its foraging grounds extend to temperate zones at high latitudes in both hemispheres (Alvarez Varas *et al.*, 2017; Dutton *et al.*, 2019). In Rapa Nui, this species is the most common in coral reefs and coastal areas and has been increasingly studied over the past decade (Álvarez-Varas *et al.*, 2015, 2021a, 2021b, 2022).

Sea turtles play a critical ecological role in maintaining the balance of marine and coastal ecosystems. In particular, green sea turtles contribute to reef resilience by grazing on algae, which prevents overgrowth and supports coral health (Wabnitz *et al.*, 2010; Goatley *et al.*, 2012). Additionally, their grazing activity is essential for sustaining the health and productivity of seagrass meadows, promoting regrowth and maintaining biodiversity (Moran and Bjorndal, 2005; Kuiper-Linley *et al.*, 2007). They also act as nutrient transporters between coastal habitats, enriching oligotrophic ecosystems through nutrient redistribution (Vander Zanden *et al.*, 2012), and serve as important prey for large sharks, integrating them into complex marine food webs (Simpfendorfer *et al.*, 2001; Heithaus *et al.*, 2002). Given their ecological significance, the conservation of green turtles is essential not only for their survival but also for maintaining the stability and health of marine ecosystems globally.

A considerable number of studies have shown that chemical pollutants can significantly impact the survival of marine populations; however, their effects on sea turtle populations remain largely unknown (Finlayson *et al.*, 2016; Arienzo, 2023; Dias *et al.*, 2024; White-Kiely *et al.*, 2024). To date, most research has focused on measuring contaminant concentrations in turtle tissues, such as blood, shell, liver, kidneys, brain and eggs, while relatively few studies have examined their impact on the health of individuals and populations (Finlayson *et al.*, 2016; Arienzo, 2023; Dias *et al.*, 2024).

Mercury (Hg) is a highly toxic heavy metal with no known biological function, which bioaccumulates and biomagnifies up the trophic webs (Day *et al.*, 2007; Rodriguez *et al.*, 2022). It occurs in three forms in the environment: elemental (metallic), inorganic (e.g. mercury salts) and organometallic (e.g. methylmercury-MeHg). Its organic form, MeHg, is of greater biological concern, as it is more efficiently absorbed and accumulated into tissues and organs than other forms of Hg, and in marine organisms enters the body mainly through their diet, constituting over 90% of total mercury (THg) (Kershaw and Hall, 2019; UNEP, 2019; Chételat *et al.*, 2020). Among its toxic effects are growth and development alterations, decreased reproductive success, impaired vision and hearing, liver and kidney damage, neurotoxicity and immunotoxicity (Day *et al.*, 2007; Perrault *et al.*, 2011). Also, it may produce widespread subtle, subclinical effects, such as neurochemical and neurobehavioral changes, changes in blood parameters, enzyme production, cardiovascular function and immune response (Boening, 2000; Basu and Head, 2010; Krey *et al.*, 2015).

Mercury is released into the atmosphere by natural emission sources such as volcanoes, geothermal vents, and Hg-enriched soil, as well as by anthropogenic activities including mine tailings, agricultural drain water, paper mills, fossil fuel burning, waste disposal and the chlorine industry (Wolfe *et al.*, 1998; Berlin *et al.*, 2007; Pérez-Rodríguez *et al.*, 2018). Anthropogenic emissions have altered mercury natural biogeochemical cycle and have increased over three times its global surface ocean water concentrations in the last century (UNEP, 2013, 2019; Obrist *et al.*, 2018). Coastal water mercury concentrations on Rapa Nui are unknown; however, a recent study reported elevated natural mercury levels in peat samples from Rano Aroi lake crater, likely originating from atmospheric deposition, and volcanic activity, among other factors (Pérez-Rodríguez *et al.*, 2018).

In 2018, the first population monitoring of the *C. mydas* aggregation in Rapa Nui was conducted. The turtles exhibited variable body condition, with some individuals appearing dehydrated and emaciated, showing signs of fishing gear interactions and lesions consistent with bacterial, fungal, and/or viral infections. Additionally, 50% of the turtles (*n* = 10) showed carapace wounds likely caused by boat propellers (Álvarez-Varas *et al.*, 2022). Hand-feeding of turtles by fishermen and tourists has been reported as a common practice on the island, primarily involving high trophic level species such as tuna (*Thunnus albacares*) and dolphinfish (*Coryphaena hippurus*) (Álvarez-Varas *et al.*, 2015). This practice may lead to behavioural changes, altered growth rates, and an increased predisposition to conditions such as obesity, malnutrition, liver and kidney diseases, diabetes mellitus, and cardiovascular disorders. Additionally, it may represent a pathway for the introduction of contaminants that bioaccumulate through the food chain (Álvarez-Varas *et al.*, 2015, 2022).

Given this background, in the present study, we analysed the demographic structure and health status of the *C. mydas* foraging aggregation in Rapa Nui (Easter Island), integrating data from 2018 and 2023. For the first time, we evaluated blood mercury concentrations and their potential impact on this endangered species on the island. In addition to providing key biological and health information, this study underscores the need to monitor a globally significant ecotoxicological pollutant on a remote South Pacific island.

## Materials and methods

### Study area and turtle capture

**Figure 1:**
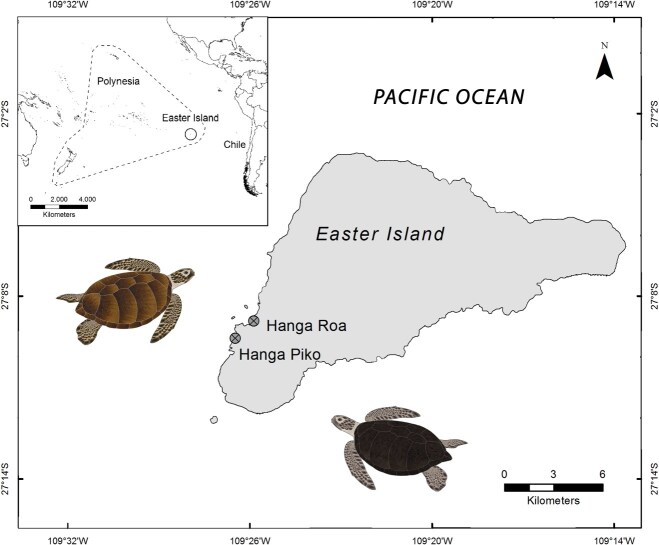
Map of the study area (Easter Island). The points indicate the locations of Hanga Roa and Hanga Piko bays, where green turtles (*C. mydas*) were sampled during 2018 and 2023

This study was carried out in the bays of Hanga Roa (27°99′S 109°269′W) and Hanga Piko (27°91′S 109°46′W), Rapa Nui (Easter Island), located in the southwest of the island, 3700 km offshore the Valparaíso Region, Chile (Fig. 1).

Two genetic lineages of *C. mydas* are present in Rapa Nui: the north-central/eastern Pacific lineage (black turtle) and the south-central/western Pacific lineage (yellow turtle) (Álvarez-Varas *et al.*, 2021a, 2021b). These lineages differ in body shape, coloration and dietary preferences (Parker *et al.*, 2011; Amorocho *et al.*, 2012; Álvarez-Varas *et al.*, 2015, 2019, 2021a). In their benthic juvenile and adult stages, black turtles are primarily omnivorous (Amorocho *et al.*, 2012; Turner Tomaszewicz *et al.*, 2018), while adult yellow turtles have been reported to feed mainly on algae and seagrasses, with invertebrates consumed to a lesser extent (Burkholder *et al.*, 2011; Prior *et al.*, 2016; Sampson *et al.*, 2018). As observed in other foraging locations (Stewart *et al.*, 2016; Turner Tomaszewicz *et al.*, 2018), both lineages (or morphotypes) in Rapa Nui are commonly provisioned by local fishers and visitors primarily using fish discards (Álvarez-Varas *et al.*, 2015, 2022).

Local trained divers performed fifty manual captures corresponding to distinct forty-five *C. mydas* individuals in the bays of Hanga Roa and Hanga Piko during November 2018 and August 2023. In all cases, turtles remained out of the water for less than 40 min. In 2018, prior to release, turtles were tagged on each front flipper using Inconel tags (National Band and Tag Company, Newport, USA) (Zárate *et al.*, 2013). In 2023, no tagging was performed due to the damage caused by the tags used previously (mainly associated with poor cicatrization and algae accumulation in the insertion area). To avoid recaptures in 2023, individuals were identified through external marks and lesions in the head, carapace and flippers. Individuals were monitored only once. Body measurements, weight and blood samples were collected. Captures were authorized by the Chilean Sub-Secretariat of Fishing (SUBPESCA, by its Spanish abbreviation), through a Research Capture Permit (Exempt Resolution N° 3755 and E-2023-492). Additionally, captures were authorized by the local community through the ‘Koro Nui o te Vaikava’ (Rapa Nui Sea Council) and the Hanga Roa and Hanga Piko fishermen’s unions.

### Morphotype, life stage and sexing

Morphotype classification was based on external characters including carapace shape, and carapace and plastron pigmentation. We assigned a yellow morphotype if the turtle had an oval carapace ranging from light to dark brown in coloration and if the plastron was cream to orange in coloration. Turtles with a domed heart-shaped carapace black to black-grey, and a greyish plastron were classified as black morphotype (Parker *et al.*, 2011; Amorocho *et al.*, 2012; Álvarez-Varas *et al.*, 2021a, 2021b, 2022).

Straight carapace length notch to tip (SCLn-t; hereafter SCL), curved carapace length notch to tip (CCLn-t; hereafter CCL) and tail total length (TTL) were measured for each turtle (Bolten, 1999). Curved and straight measurements were obtained using a metric tape and a calibrated forester’s calliper (0.1 cm, straight measurements), respectively. Body mass was obtained using a digital scale (± 0.1 kg).

The individual’s life stage (juvenile/adult) was based on the putative natal origin of each morphotype according to Álvarez-Varas *et al.* (2022), corresponding to Galapagos for black turtles and French Polynesia for yellow turtles. In both cases, the cutoff was set at 85 cm CCL, as this is the average CCL of nesting females in both the Galapagos and French Polynesia. Thus, individuals with CCL < 85 cm were classified as juveniles and CCL ≥ 85 cm as adults (Zárate *et al.*, 2013; Dolfo *et al.*, 2023). Turtles with TTL ≥ 25 cm were classified as males (McFadden *et al.*, 2014).

### Physical examination and BCI estimation

Physical examination was carried out by a veterinarian following the protocol detailed in Álvarez-Varas *et al.* (2022). Briefly, the examination involved a visual assessment from proximal to caudal, inspecting the eyes, ears, nasal cavity and oral cavity for lesions, trauma or foreign materials. The carapace and plastron were evaluated for firmness, injuries and epibionts, which were removed if possible. Mobility, muscle tone, and flipper strength were assessed, along with any signs of inflammation, deformities or damage. The tail and cloaca were checked for foreign materials, masses or prolapse. Furthermore, a body condition index (BCI = [body mass/SCLn-t3] × 10 000) (Bjorndal *et al.*, 2000; Koch *et al.*, 2007; Álvarez-Varas *et al.*, 2021b) was calculated to evaluate the relative “fatness” of turtles. This index was used as an indirect predictor of the nutritional status and/or health condition of animals (Koch *et al.*, 2007; Alvarez Varas *et al.*, 2017).

### Blood count and blood chemistry analyses

Blood samples were collected from the dorsal cervical sinus and stored at refrigeration (4°C) or freezing (−20°C) temperatures depending on the analysis. In both cases, the cold chain was maintained using dry ice from Easter Island to the laboratory. Samples for blood count (4 ml) were stored in heparinized tubes (sodium heparin) at 4°C. The complete blood count was performed by manual counting. The packed cell volume (PCV%) was obtained using a microhaematocrit tube, where capillaries were prepared with ¾ of heparinized blood and centrifuged for 5 min at 5000 rpm (2800 g). Results were obtained by measurement of each capillary. Haemoglobin (HB) was measured using a haematology analyser (Mindray BC. 2800 VET). The erythrocyte count (red blood cells, RBC) was performed manually using modified Neubauer haemocytometer chamber (central grid of 5 × 5 lines), with a Natt and Herrick solution as diluent. Mean corpuscular volume (MCV), mean corpuscular haemoglobin (MCH) and mean corpuscular Hb concentration (MCHC) were obtained according to Latimer (2011). For the absolute leukocyte count (white blood cells, WBC), manual counting with a modified Neubauer haemocytometer chamber and a Natt and Herrick diluent was also used. The differential count of leukocytes was performed from a blood smear (100 leukocytes with differentiation of heterophils, lymphocytes, eosinophils, basophils, monocytes and azurophils). Thrombocytes were also counted using a blood smear.

For blood chemistry analyses, blood (4 ml) was stored in a tube without anticoagulant. Two ml of blood was centrifuged at 5000 rpm (2800 g) for five min (Nuve nf 200 centrifuge) within 12 h after collection to obtain serum. Serum was stored at −20°C for five days until analysis. The remaining blood (2 ml) was frozen at −20°C for mercury determination. Serum analytes, including alanine aminotransferase (ALT), alkaline phosphatase (ALP), aspartate aminotransferase (AST), calcium (CA), cholesterol (CHOL), creatine phosphokinase (CPK), creatinine (CREA), gamma glutamyl transferase (GGT), phosphorus (PHOS), total protein (TP), urea nitrogen (BUN), uric acid (URIC AC), albumin (ALB), globulin (GLOB) and urea, were measured using a wet chemical analyser (Mindray BS-200) following the manufacturer’s specifications, warranty and quality control (QC). All analyses were carried out at Laboratorio Clínico Veterinario SpVet, Buin, Santiago, Chile.

### Blood THg determination

From frozen blood without anticoagulant (2 ml), a total of 20 mg of clot was analysed using thermal decomposition, amalgamation and atomic absorption with a double beam Direct Mercury Analyser (DMA-evo80, Milestone, Sorisole (BG)—Italy). The EPA Method 7473, where no pretreatment of samples is needed for THg determination (Hg henceforth), was developed (EPA, 1998). Mercury concentrations were expressed in ppb wet weight (w.w.).

The quality assurance and QC (QA/QC) included blanks every ten samples. Certified references materials (DORM-5, fish protein certified reference material, National Research Council Canada) was used at the beginning and end of each batch of samples analysed, to confirm that sample combustion and minimize contamination, and random duplicates to confirm minimum variability between samples. Limit of detection (LoD) of the instrument is 0.0003 ng Hg; LoD and limit of quantitation (LoQ) were 0,0022 and 0.0065 ng, respectively. Reference material recovery average 88.43% ± 3.5 (SE; range 84.96–91.91%). Final concentration was corrected using blanks and percentage of recovery for each batch of samples (EPA, 1998). Analyses were carried out at the Laboratory of Biogeochemistry of Contaminants and Aquatic Ecotoxicology (BEA), Universidad Andrés Bello, Santiago, Chile.

### Statistical analysis

Means, standard deviations (SD) and ranges were calculated for morphological data, BCI, haematological parameters (blood count and blood chemistry analyses) and blood Hg concentrations. After verifying the normal distribution of the data using the univariate normality test (Anderson-Darling) and Levene’s test, a parametric analysis of variance (ANOVA) was performed to assess differences between morphotypes, life stages, and years for SCL and BCI (given that both are comparable measurements related to the size and condition of the animals). Statistical analyses for this section were conducted using the car, nortest and vegan libraries. Additionally, a Student’s *t*-test was used to analyse differences in BCI between turtles with and without carapace damage. The results were graphically represented using a box plot created with the ggplot2 library.

For blood Hg concentrations and their relationship with haematological variables, the variance inflation factor (VIF) was used to identify and mitigate collinearity issues among the analytes and Hg concentrations (Zuur *et al.*, 2010). The resulting variables were subjected to the Shapiro–Wilk normality test. Subsequently, a multivariate analysis of variance based on permutations (PERMANOVA) was performed, using Gower’s distance as the dissimilarity metric and 999 permutations to assess the statistical significance of the associations between haematological variables and mercury levels. Additionally, an exploratory heatmap was generated to visualize potential correlations between the variables resulting from the VIF analysis, blood mercury concentrations, SCL and BCI. Since more than half of the variables did not follow a normal distribution, Spearman’s correlation was used to assess relationships between them. The heatmap was created using the ggplot2, reshape2 and Hmiss libraries. All statistical analyses were conducted in RStudio version 2024.12.0.467.

### Ethical statement

All procedures were approved by the Scientific Ethical Committee of the Universidad Católica del Norte, Coquimbo, Chile (CEC UCN N°15, 2021).

## Results

### Morphotype, life stage and sexing

A total of 20 *C. mydas* individuals were captured in 2018, 15 (75%) corresponded to the yellow morphotype and five (25%) to the black. In 2023, 30 turtles were captured on the island, 23 (76.7%) were yellow turtles and seven (23.3%) were black. Of the total captured in both periods, 45 were new individuals while five were recaptures; three of them were yellow turtles and two were black. One turtle (classified as black morphotype) was not included in the posterior analysis due to missing morphological and haematological data. For the recaptures, the morphometric variables (including mass and BCI) were averaged between the two years. Details on measurements and biological data of each morphotype are shown in Table 1.

**Table 1:** Morphometric data, mass and BCI (mean ± SD) of both *C. mydas* morphotypes from Rapa Nui captured in 2018 and 2023

Parameter	Yellow morphotype (*n* = 35)		Black morphotype (*n* = 9)		All (*n* = 44)	
	Mean ± SD	Range	Mean ± SD	Range	Mean ± SD	Range
SCL	57.20 ± 12.76	40.00–91.50	67.13 ± 11.64	54.80–87.00	59.53 ± 13.10	40.00–91.50
CCL	61.92 ± 14.69	43.10–102.00	76.23 ± 11.79	60.30–93.50	65.03 ± 15.14	43.10–102.00
TTL	11.42 ± 4.35	7.40–30.40	14.56 ± 3.43	10.40–20.00	12.25 ± 5.19	7.40–33.30
Mass	33.77 ± 27.22	9.30–138.00	60.88 ± 30.64	21.50–107.00	39.43 ± 30.02	9.30–138.00
BCI	1.66 ± 0.26	1.11–2.37	1.67 ± 0.21	1.28–1.90	1.66 ± 0.25	1.11–2.37

**Figure 2:**
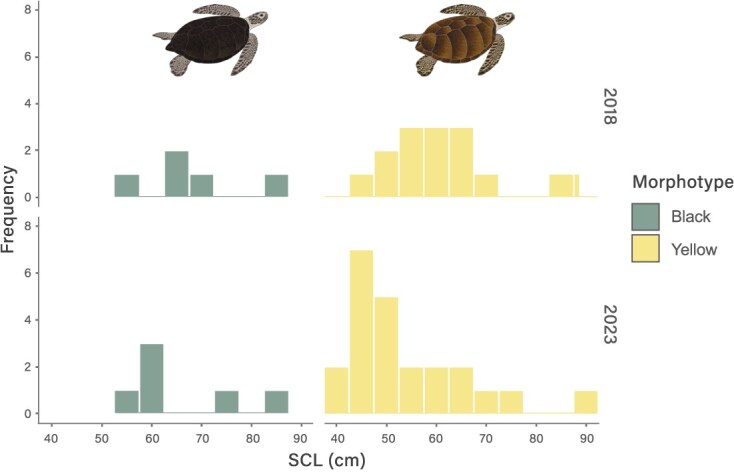
Histogram of the straight carapace length notch to tip (SCL) for both Pacific *C. mydas* morphotypes from Rapa Nui in 2018 and 2023

In 2018, 16 turtles (80%) were classified as juveniles and four as adults (20%, three females, one male). In 2023, 25 individuals (83.3%) corresponded to juveniles and four to adults (16.7%, two females, two males). Overall, for both periods and both morphotypes, the study area was dominated by small turtles with individuals varying between 40 and 70 cm SCL (Fig. 2). The SCL varied between 40.0 and 91.50 cm (mean of 59.53 ± 13.10 cm) with weights between 9.3 and 138.0 kg (mean of 39.43 ± 30.02 kg; Table 1).

Yellow turtles were significantly smaller than black ones (*P* = 0.006837) and individuals from 2018 were significantly larger than those from 2023 (*P* = 0.0003774), regardless of the morphotype (Fig. 2). Nevertheless, the interaction between morphotype/year was not significant (*P* = 0.197811).

### Physical examination and BCI

In 2018, physical examination of turtles showed variable body condition with some turtles apparently dehydrated and emaciated, exhibiting signs of fishing gear interaction, and lesions congruent with bacterial, fungal and/or viral infections. No epibionts on carapace nor skin were observed (see Álvarez-Varas *et al.*, 2022 for further details). Fifty percent of sampled individuals (*n* = 10) had carapace lesions likely due to boat collisions (Álvarez-Varas *et al.*, 2022). In 2023, turtles also had variable body condition, without presence of epibionts (but with previous epibiont scars), nor fishing gear interaction signs. Nevertheless, eight individuals (26.7%, *n* = 30) showed carapace scars, and one of them exhibited a deep fracture in the posterior carapace region resulting in severe buoyancy alteration. On the other hand, a turtle who exits the water every day for sunbathing in Hanga Piko bay (Fig. 1) was examined. This turtle had deep, healed lesions on its carapace, poor body condition with a high amount of body fat in the neck, flippers, and inguinal area, and numerous lesions and unhealed wounds on its soft parts (including the head). Most small juveniles (ranging from 40.8 to 47.3 cm SCL, *n* = 5) exhibited a large amount of algae on their carapace, plastron and flippers; they appeared to be dehydrated and visibly showed very poor body condition. Four individuals from 2023 (13.3%) exhibited lesions on head, eyes, flippers and cloaca, congruent with infectious agents.

**Figure 3:**
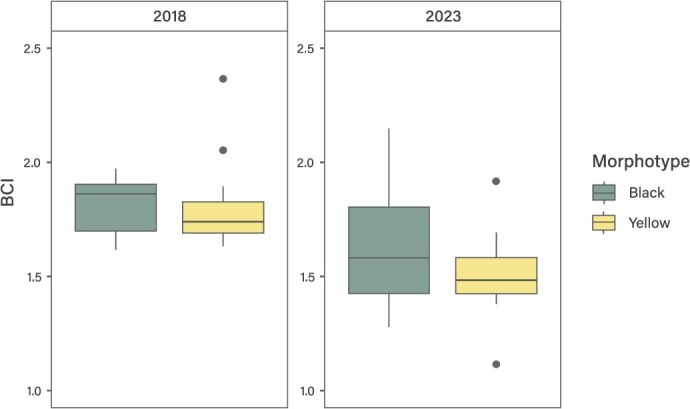
Box plots of the body condition index (BCI) for both Pacific *C. mydas* morphotypes from Rapa Nui in 2018 and 2023

BCI ranged between 1.11 and 2.37 (mean of 1.66 ± 0.26) and between 1.28 and 1.90 (mean of 1.67 ± 0.21) for yellow and black turtles, respectively. For turtles from 2018 and 2023, BCI ranged between 1.62 and 2.37 (mean of 1.81 ± 0.19) and between 1.11 and 2.15 (mean of 1.54 ± 0.22), respectively. Significant differences were found in BCI between years (*P* = 0.0003774; Fig. 3) and for the interaction between morphotype/life stage (*P* = 0.02637).

**Figure 4:**
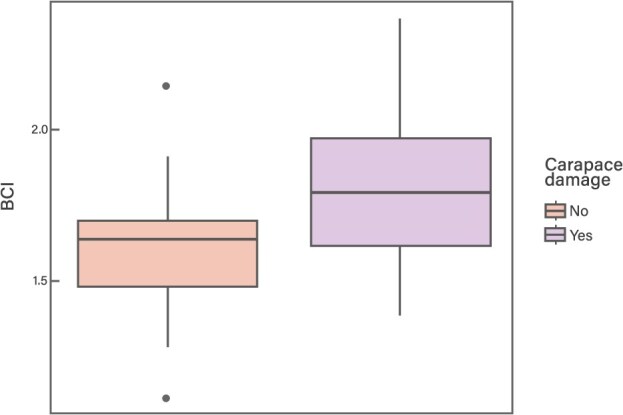
Box plots of the body condition index (BCI) for *C. mydas* individuals with and without carapace damage from Rapa Nui in 2018 and 2023

When comparing the BCI between turtles with carapace damage and those without (*n* = 14, including both study years), significant differences were found between the groups (*P* = 0.03814; Fig. 4), with higher BCI values in the turtles with damage (mean of 1.79 ± 0.29 and 1.60 ± 0.22 for turtles with and without carapace damage, respectively).

### Haematological analyses, mercury concentrations and correlation between biological parameters

Blood count analyses (*n* = 14) showed only four individuals (28.6%) had normal erythrocyte (red series) and leukocyte (white series) morphology. Forty-three percent of turtles (*n* = 6) exhibited a PCV%, RBC and HB concentration below limits reported for the species (Table 2). These individuals were all juveniles and showed higher levels of MCV and MCH, indicating macrocytic hyperchromic anaemia (Stacy *et al.*, 2011).

**Table 2:** Blood count and blood chemistry (mean ± SD) of *C. mydas* individuals from this study and other published studies in the Pacific Ocean

Parameter	This study		Fernández-Sanz *et al.* (2023)	Howell and Shaver (2021)	Banerjee *et al.* (2021)	March *et al.* (2018)	Reséndiz *et al.* (2018)	Alvarez Varas *et al.* (2017)	Suarez-Yana *et al.* (2016)	McFadden *et al.* (2014)	Komoroske *et al.* (2011)	Flint *et al.* (2010)	Labrada-Martagón *et al.* (2010)
	Mean ± SD	Range											
Blood count (*n* = 14)													
PCV%	28.57 ± 7.50	14.28–37.09	38.00	37.15	34.70	23.00	39.60	34.00	33.00	32.2	38.19	33.30	
RBC 10^6^/ul	1.32 ± 0.51	0.49–1.97	0.49				3.12		0.52				
HB g/dl	10.15 ± 2.80	5.00–13.80	11.80	11.49			8.72						
MCV fl	229.26 ± 37.27	180.70–291.40					61.91		716.51				
MCH pg	81.36 ± 15.10	64.20–111.40					19.1						
MCHC g/dl	35.53 ± 3.72	31.08–44.14		30.59			11.82						
THROMB K/ul	37.93 ± 24.00	10.00–89.00					18.76					75.00	
LYMPH ul	2798.14 ± 1027.48	1197.00–4394.00	1900.00	117.50		2800.00	6330.00		2140.00	2782.00	7251.00	11450.00	
HETEROPH ul	6577.93 ± 3595.86	2296.00–14240.00	4530.00	498.60		13200.00	6230.00		6690.00	3951.00	2668.00	4950.00	
EOSINOPH ul	292.14 ± 258.71	0.00–856.00	650.00	45.55			1240.00		120.00	1313.00	1756.00		
MONOCYT ul	489.57 ± 263.58	41.00–1014.00	940.00	26.06		1400.00	1830.00		910.00	476.00	566.30	2520.00	
BASOPH ul	45.00 ± 69.99	0.00–214.00		78.80					130.00		85.38		
AZUROPH ul	12.07 ± 45.17	0.00–169.00											
WBC ul	10210.71 ± 4064.58	4100.00–17800.00	8030.00	810.00		17900.00	8150.00		9980.00		11970.00	20850.00	
*Blood chemistry* (*n* = 21)													
AST U/L	161.74 ± 76.24	19.10–280.40	146.00	184.80	180.00	192.70	250.15	298.00	191.17	186.00	153.30	159.35	191.39
ALT U/L	16.79 ± 24.02	6.40–119.20	4.00	3.97			34.15		32.38		5.50		18.84
ALP U/L	7.92 ± 9.33	0.80–42.00	58.00	316.00					38.04	24.00	73.05	23.10	42.63
GGT U/L	2.35 ± 8.78	0.00–40.10	2.00										1.75
CHOL mg/dl	84.55 ± 50.30	6.00–195.20	146.33	163.60	208.00					164.90	228.60		164.80
CREA mg/dl	0.08 ± 0.04	0.00–0.10	0.31	0.43	0.10		0.62	0.37	0.25			0.41	1.97
BUN mg/dl	30.74 ± 33.01	1.90–103.60		4.10	15.10		10.50				22.38		
UREA mg/dl	65.84 ± 70.76	3.70–222.00	59.64				24.30		64.31			82.60	
CA mg/dl	5.98 ± 1.38	4.00–9.00		7.38	8.60			10.30		9.70	8.24	4.81	7.52
PHOS mg/dl	4.71 ± 3.33	0.80–16.70	12.59	8.66	8.70			12.46		5.10	8.98	8.06	11.26
TP g/dl	3.6 ± 1.63	1.30–8.90	5.10	4.35	4.90	2.39	5.03	5.90	4.20	4.10	5.81	4.15	5.46
ALB g/dl	1.46 ± 1.05	0.30–5.50		1.79	1.80		1.53	2.45		1.30	2.16	1.22	1.26
GLOB g/dl	2.14 ± 0.77	0.70–3.50		2.54			3.46	3.45		2.80	3.65	3.10	4.28
URI AC mg/dl	1.27 ± 2.18	0.10–10.50	1.30	1.21	1.30	0.00				0.90	1.08	1.29	2.64
CPK U/L	1981.60 ± 2137.62	243.50–10081.00	715.00		955.00	4439.11		2479.00		1045.00	1312.00		

Some of them also presented polychromatic erythrocytes (immature red blood cells), anisocytosis (red blood cells that vary in size) and poikilocytosis (red blood cells with abnormal shapes); one exhibited azurophils, and all of them exhibited the presence of toxic heterophils. Overall, the number of lymphocytes were below those reported for healthy green turtles (Table 2) and were similar to unhealthy individuals (Table 2; March *et al.*, 2018). Values of eosinophils and basophils were below those reported in other studies and heterophiles, monocytes and WBC were within range. However, in many cases, the intervals were widely variable (Table 2).

Blood chemical analyses (*n* = 21) resulted in low levels of ALP, cholesterol, creatinine, calcium, phosphorus, total protein and globulins (Table 2). Values of BUN and CPK were above those reported in most green turtle studies (Table 2). The rest of parameters were within range reported for the species (Table 2). Mean values, SD and range of haematological parameters are shown in Table 2.

VGA levels were correlated with VCM (r^2^ = −0.776; *P* = 0.001), and CHCM was correlated with cholesterol (r^2^ = 0.657; *P* = 0.011). Basophil values were correlated with eosinophils (r^2^ = 0.723; *P* = 0.003), calcium (r^2^ = 0.698; *P* = 0.006), creatinine (r^2^ = −0.579; *P* = 0.030) and BCI (r^2^ = 0.693; *P* = 0.026). Monocytes were correlated with AST (r^2^ = 0.666; *P* = 0.009), AP (r^2^ = 0.643; *P* = 0.013), and lymphocytes (r^2^ = 0.604; *P* = 0.022). Lymphocytes were also correlated with AP (r^2^ = 0.623; *P* = 0.017). Eosinophil and heterophil values were correlated with calcium (r^2^ = 0.564; *P* = 0.036 for eosinophils and r^2^ = −0.707; *P* = 0.005 for heterophils). Eosinophils were also correlated with BCI (r^2^ = 0.869; *P* = 0.000).

Among the biochemical profile analytes, UREA levels were correlated with calcium (r^2^ = −0.558; *P* = 0.009), GGT (r^2^ = −0.598; *P* = 0.004), AST (r^2^ = 0.583; *P* = 0.006) and BCI (r^2^ = −0.579; *P* = 0.019). AST was also correlated with ALP (r^2^ = −0.579; *P* = 0.006) and globulins (r^2^ = 0.458; *P* = 0.037). GGT values were correlated with SCL (r^2^ = −0.468; *P* = 0.032) and BCI (r^2^ = 0.525; *P* = 0.037). Globulins were correlated with cholesterol (r^2^ = 0.442; *P* = 0.045), creatinine with calcium (r^2^ = −0.435; *P* = 0.049), and ALP with CPK values (r^2^ = 0.447; *P* = 0.042). Cholesterol values were also correlated with SCL (r^2^ = 0.505; *P* = 0.019).

**Figure 5:**
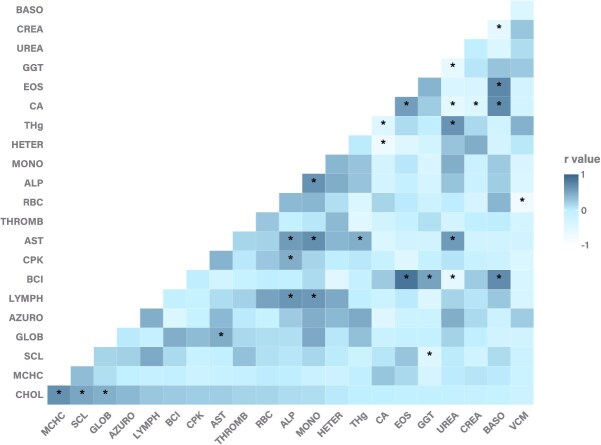
Heatmap representing the relationship between straight carapace length notch to tip (SCL), body condition index (BCI), haematological parameters and blood mercury concentrations (Hg) in *C. mydas* from Rapa Nui. ^*^ *P- values* ≤ 0.05

Although most individuals exhibited Hg levels below limits of detection (*n* = 14); seven turtles showed concentrations of this nonessential element in blood. The mean value of Hg was 93.03 ± 53.77 ppb w.w. (SE) with a range between < LoQ and 338.21 ppb w.w. (Table 3). All these turtles were juveniles, six of them were yellow and one was black. Given the small sample size (*n* = 7), correlation analyses between SCL, BCI, haematological parameters, and mercury levels were performed without considering morphotypes or life stage. Mercury levels were positively correlated with UREA (r^2^ = 0.639; *P* = 0.009) and AST (r^2^ = 0.450; *P* = 0.041) and negatively correlated with calcium (r^2^ = −0.465; *P* = 0.034) (Fig. 5).

**Table 3:** Mercury concentrations (mean ± SD) in blood (clot) of *C. mydas* individuals from this study and other published studies worldwide

Habitat	Life stage	*n*	Tissue	Hg ± SD (ppb)	Hg range (ppb)	Location	Reference
Foraging ground	Juvenile/adult	21	Clot	93.03 ± 53.77 (SE)	< LOQ338.21	Rapa Nui, Chile	This study
Foraging ground	Juvenile	33	Whole blood	8.00	0.00–245.00	Itapirubá North beach, Brazil	Pérez *et al.* (2023)
Foraging ground	Juvenile	18	Whole blood	4.00	0.00–39.00	Cassino beach, Brazil	
Foraging ground	Juvenile	69	Whole blood	0.21 ± 0.17	0.01–0.99	Coroa Vermelha, Brazil	Miguel *et al.* (2022)
Foraging ground	Juvenile	55	Whole blood	0.11 ± 0.09	0.02–0.59	Santa Cruz, Brazil	
Foraging ground	Juvenile/adult	36	RBC	< LOQ		Southern California, USA	Barraza *et al.* (2019)
Foraging ground	Juvenile/adult		Whole blood	< LOQ		Queensland, Australia	Villa *et al.* (2019)
Foraging ground	Juvenile	9	RBC	3.00	1.00–50.00	Gulf of Mexico, USA	Perrault *et al.* (2017b)
Foraging ground	Juvenile	21	Whole blood	30.00–10.00		Cape verde	Camacho *et al.* (2014)
Foraging ground	Juvenile	12	Cardiac blood	8.00 ± 1.00		Texas, USA	Faust *et al.* (2014)
Foraging ground	Juvenile/adult	54	Whole blood	1.90 ± 3.80	1.00–24.90	Palmyra Atoll, Pacific Ocean	McFadden *et al.* (2014)
Foraging ground	Juvenile	40	Plasma	9.30 ± 1.80	0.22–38.00	Gladstone, Australia	Gaus *et al.* (2012)
Foraging ground	Juvenile/adult	15	RBC	2.13 ± 0.42		San Diego Bay, USA	Komoroske *et al.* (2011)
Foraging ground	Juvenile/adult	30	Whole blood	1.01 ± 0.16			
Foraging ground	Adult	16	Whole blood	2.51 ± 0.50	0.25–7.12	Gold Coast, Queensland, Australia	van de Merwe *et al.* (2010)
Foraging ground/Nesting	Juvenile/adult	3	Whole blood	< LOQ		Southern Turkey	Yipel *et al.* (2017)
Nesting	Adult	60	Whole blood	1.16		Quintana Roo, Mexico	Mondragón *et al.* (2023)
Nesting	Adult	27	Whole blood	6.00		Gulf of Guinea	Morão *et al.* (2022)
Nesting	Adult	12	Whole blood	160.00 ± 40.00		Sea of Oman	Sinaei and Bolouki (2017)
Stranded	Juvenile	12	Serum	< LOQ		Gulf of Thailand	Chomchat *et al.* (2023)
Stranded	Juvenile	3	Serum	< LOQ		Andaman Sea	Chomchat *et al.* (2023)

## Discussion

*Chelonia mydas* play a crucial role in marine ecosystems but are highly vulnerable to threats such as pollution (Finlayson *et al.*, 2016; Dias *et al.*, 2024). Mercury pollution, though a significant concern, remains understudied in relation to the health and survival of this species (Komoroske *et al.*, 2011; Perrault *et al.*, 2017a; Rodriguez *et al.*, 2022). Investigating the effects of Hg across different regions is critical not only for developing effective conservation strategies but also for understanding the broader impacts of environmental contamination on marine biodiversity, ecosystems and public health (Landrigan *et al.*, 2020). Furthermore, understanding the demographic structure and health of sea turtles is essential for designing targeted, locally adapted management strategies, particularly for endangered species like *C. mydas* (Joseph *et al.*, 2023).

This study examines the *C. mydas* aggregation in Rapa Nui, a remote area where anthropogenic pressures intersect with unique environmental factors (Álvarez-Varas *et al.*, 2015, 2022). Our findings offer valuable insights into the health and demographic characteristics of this group, which are crucial for local species management strategies. They also emphasize the need for systematic Hg monitoring in the environment, turtles and the Rapa Nui human population.

### Potential factors influencing turtle arrival to Rapa Nui waters

Rapa Nui *C. mydas* aggregation is composed of juveniles and adults of both Pacific morphotypes (Álvarez-Varas *et al.*, 2021a, 2021b), with dominance of yellow morphotype turtles. A recent study of this species on the island suggested that the most likely natal origin of the yellow morphotype is French Polynesia, located ~4200 km from Rapa Nui. Black turtles probably come from Galapagos, Ecuador, situated ~3580 km away (Álvarez-Varas *et al.*, 2022). Several studies have proposed that foraging area selection by adult and juvenile sea turtles is influenced by factors such as relative size of nesting populations, distance between nesting and foraging locations, surface water temperature, and offshore currents (Lahanas *et al.*, 1998; Luke *et al.*, 2004; Jensen *et al.*, 2020). In the South Pacific Ocean, the main current is the counter-clockwise South Pacific Subtropical Gyre, comprising four prevailing counter-clockwise currents: the northern South Equatorial Current, the western East Australian Current, the southern South Pacific Current and the eastern Humboldt Current (Schneider *et al.*, 2007). This circular Gyre has numerous mirroring currents that circle downward within the main Gyre (Glynn *et al.*, 2003; Schneider *et al.*, 2007). Although the straightline distance from Galapagos to Rapa Nui is less than from French Polynesia, these local currents within the South Pacific Gyre could be facilitating the arrival of yellow turtles from French Polynesia to Rapa Nui to a larger extent. Black turtle dispersal from Galapagos also could be influenced by this main current system (South Pacific Gyre), but given its counter-clockwise direction, turtle displacement from this point would be slower and likely take longer, which would justify the lesser presence of this morphotype at the island. Factors such as the relative size of nesting populations contributing individuals to Rapa Nui, surface water temperatures and local surface currents should be studied in detail in the future to understand how they influence the arrival of turtles to this remote island in the South Pacific.

### Dominance of small juveniles and ENSO influence: new arrivals or winter dormancy?

Both study periods (2018 and 2023) were conducted during El Niño events with warmer surface waters (Santidrián Tomillo *et al.*, 2020), which could explain the arrival of turtles of variable sizes to the island (Quiñones *et al.*, 2010; Alvarez Varas *et al.*, 2017). With the same capture effort (eight days, eight hours per day and two trained divers capturing all turtles accessing Hanga Roa bay), an apparently higher abundance of turtles was detected in August 2023 (*n* = 30) than in November 2018 (*n* = 20). This could be related to a strong El Niño phase in 2023 and by contrast, a weak El Niño event in 2018, which should be studied in further detail.

In August 2023, we captured a higher number of small juveniles than in 2018. Sizes were between 40.8 and 47.3 cm SCL; they exhibited their heads, carapaces and flippers fully covered in algae and visually showed dehydration and poor body condition. Furthermore, most of these small juveniles exhibited altered haematological values with macrocytic-hyperchromic regenerative anaemia, and presence of azurophils and toxic heterophils, suggestive of ongoing inflammatory process or infectious disease (Stacy *et al.*, 2011, 2018). Small turtles with these characteristics may suggest that they recently recruited to the island, as other studies have reported that newly arrived juvenile turtles to coastal foraging areas from the open ocean often are emaciated, covered in barnacles, and in poor health and body condition (Kubis *et al.*, 2009; Pilcher *et al.*, 2015).

Sampson *et al.* (2014) studied the size distribution and body condition of black and yellow turtles from a foraging aggregation in the Colombian Pacific. As in our study, the size of recruitment in Colombia was 40.0 to 49.9 cm SCL for both morphotypes, with more yellow than black turtles in this size class, indicating a difference in the recruitment pattern. Likewise, minimum size of turtle recruit into Rapa Nui coastal foraging ground (~40 cm SCL) is similar to other Pacific aggregations such as Australia (40 to 50 cm CCL; Limpus, 2008); Hawaii (~35 cm SCL; Arthur and Balazs, 2008), and other Central, West and South Pacific islands (~35 cm SCL; Becker *et al.*, 2019); but higher than Fijian turtles, which recruit starting at ~25 cm CCL (Piovano *et al.*, 2020).

On the other hand, given the season (August 2023, austral winter), it is possible that turtles with such characteristics (fully covered in algae, dehydrated, with poor body condition and altered haematological values) have just come out of a brumation period. During brumation, also called “winter dormancy,” turtles reduce their activity, movements, and metabolism to tolerate low water temperatures (Felger *et al.*, 1976; Vélez-Rubio *et al.*, 2017, 2022). During this phenomenon carapaces are often covered with benthic community biota such as barnacles, hydrozoans, seaweed, mussels and polychaetes (Reyes *et al.*, 2020; Vélez-Rubio *et al.*, 2022). Most studies on brumation in green turtles have reported that it occurs at SST below 12°C (Felger *et al.*, 1976; Vélez-Rubio *et al.*, 2017); however, sea surface temperatures in Easter Island range from 19 to 25°C (Glynn *et al.*, 2003). Stable isotope studies based on differences in δ13C/δ15N values to differentiate resident versus recently recruited turtles (Piovano *et al.*, 2020), and analysis of local benthic hard bottom invertebrates and algae (Vélez-Rubio *et al.*, 2022) could provide insights into whether these small individuals are new arrivals or they are coming out of winter dormancy.

### Possible anthropogenic influences on the body condition of Rapa Nui turtles with carapace damage

In both study periods, we identified turtles with carapace damage of varying severity (ranging from superficial wounds to deep fractures) and stages of healing (recent or fully cicatrized injuries). Most lesions appeared to result from boat propeller strikes, as evidenced by ruptures, deformations and parallel straight cuts or deep scratches on the carapace (Denkinger *et al.*, 2013; Himpson *et al.*, 2023). Boat strikes are a well-documented source of anthropogenic injuries in marine turtles, especially in regions with high levels of boat traffic (Barco *et al.*, 2016; Himpson *et al.*, 2023) such as Rapa Nui (Álvarez-Varas *et al.*, 2015, 2022).

Our results show significant differences in BCI between turtles with carapace damage and those without, with higher BCI values observed in the former group. In cases of acute trauma due to boat strikes, it is likely that affected animals die immediately, and postmortem examinations do not reveal significant health alterations, often showing good body condition (Barco *et al.*, 2016). In contrast, turtles that survive the impact are likely to experience disruptions in normal diving and foraging behaviour, leading to anorexia and subsequent declines in body condition (Work *et al.*, 2015; Barco *et al.*, 2016; de Mello and Alvarez, 2020).

The highest BCI exhibited by Rapa Nui turtles showing carapace damage may be due to reduced mobility, leading to prolonged stays in the bay where they have access to artificial feeding provided by fishermen and visitors (Álvarez-Varas *et al.*, 2015, 2022; Stewart *et al.*, 2016). In fact, several turtles with carapace damage had very high BCI values (greater than 2.0). Although it is not possible to determine if these values are anomalous, as they are specific to each population, the general BCI averages for healthy *C. mydas* Pacific populations range between 1.35 and 1.66 (1.42, Seminoff *et al.*, 2003; 1.35, Koch *et al.*, 2007; 1.5, Velez-Zuazo *et al.*, 2014; 1.64, Fukuoka *et al.*, 2015; 1.66, Alvarez Varas *et al.*, 2017; 1.4, Finlayson *et al.*, 2022). Additionally, the study by Stewart *et al.* (2016) compared a group that received supplemental feeding as part of a tour off the coast of Barbados with an unsupplemented group and observed that turtles in the supplemented group had a higher BCI (1.4 vs 1.2 in supplemented vs unsupplemented turtles). This suggests that food subsidies in the bays may be negatively impacting the body condition of injured animals, potentially leading to malnutrition or excessive weight. A high-protein diet, combined with easily accessible calorie sources, could contribute to obesity and increase susceptibility to conditions such as hepatic lipidosis (Stewart *et al.*, 2016). Further research involving a larger sample of affected turtles will help confirm the pattern observed in this study. Expanding the study to include more turtles in Rapa Nui will allow assessment of the impact of carapace damage on haematological parameters, as well as other health indicators, which could not be evaluated due to the small sample size (*n* = 3 turtles with carapace damage and available haematological data).

### Haematological analyses suggest compromised turtle health associated with environmental stressors

In 2018, adults and juveniles exhibited lesions on their head, eyes, flippers and cloaca consistent with infectious diseases (Álvarez-Varas *et al.*, 2022), prompting health monitoring through blood analyses of this turtle aggregation in 2023. Blood count analysis of Rapa Nui turtles revealed lymphopenia, characterized by a low count of eosinophils and basophils (Table 2). Factors such as infectious diseases and nutritional deficiencies are known to contribute to lymphopenia (Aguirre *et al.*, 1995; Montilla Fuenmayor *et al.*, 2006; Steinberg and Divers, 2020). Additionally, low lymphocyte counts may also be linked to mercury exposure, which has been shown to suppress immune function in loggerhead turtles (Day, 2003; Day *et al.*, 2007). Eosinopenia, on the other hand, could result from variations in diet and parasite load, as observed in loggerhead and green sea turtles (Stacy *et al.*, 2011). However, these findings, along with the observed basopaenia, could also be related to brumation periods in reptiles (Strik *et al.*, 2007).

Labrada-Martagón *et al.* (2010) suggested that elevated levels of triglycerides, total protein, albumin and globulins, coupled with good body condition in turtles, could indicate a food-rich environment. Similarly, de Mello and Alvarez (2020) found that green turtles in good body condition exhibited higher serum levels of albumin, cholesterol, and phosphorus compared to those in fair body condition. In contrast, low concentrations of calcium, potassium and phosphorus in turtle blood samples may signal poor health within a foraging aggregation (Labrada-Martagón *et al.*, 2010). Blood chemistry analyses of Rapa Nui turtles revealed low levels of ALP, cholesterol, creatinine, calcium, phosphorus, total protein and globulins (Table 2). These findings may reflect limited food availability or poor food quality in the region (Labrada-Martagón *et al.*, 2010). Alternatively, the combination of these results with low RBC and HB levels may indicate diseases or injuries that impair food intake, leading to poor nutritional status (de Mello and Alvarez, 2020). Abnormalities in both white and red blood cell lines (including lymphopenia, azurophils, eosinopenia, basopaenia, anaemia and toxic heterophils), along with reduced calcium, phosphorus and globulin levels and elevated CPK and BUN, suggest an ongoing inflammatory and/or infectious process (Labrada-Martagón *et al.*, 2010; de Mello and Alvarez, 2020). These findings are consistent with the carapace lesions and overall health status observed in several individuals analysed in this study.

### Potential sources of mercury pollution affecting Rapa Nui turtles

Despite variations in methodology and blood matrices (e.g. clot, RBC, whole blood), our results show higher blood mercury concentrations (93.03 ppb; Table 3) than most global studies on *C. mydas*, except for green turtles nesting on the northern coast of the Sea of Oman, which had Hg levels of 160 ppb (Sinaei and Bolouki, 2017; Table 3). Differences in mercury concentrations between species and populations are likely due to differences in diet and/or local feeding (Perrault *et al.*, 2017a). For instance, studies have reported elevated Hg concentrations in Georgia loggerheads, whose foraging ground is geographically close to a superfund site and a coal power plant (Day *et al.*, 2005; Deem *et al.*, 2009). A similar situation could be occurring on the northern coast of the Sea of Oman, where heavy metals are discharged into the marine ecosystem from industrial and development projects (Sinaei and Bolouki, 2017).

Rapa Nui is in the southeastern Pacific region where multiple geological hotspots and active hydrothermal vents exist (Hekinian *et al.*, 1996; Gueguen *et al.*, 2022). Only in the area around Rapa Nui and Salas y Gómez islands, there are over 3000 submarine volcanic structures (Rappaport, 1997). Although no studies on Hg levels in coastal waters around the island have been published, a recent investigation analysed Hg concentrations in peat and vegetation samples from Rano Aroi crater lake, and higher values than those recorded in most peat records belonging to the industrial period were found, highlighting that natural factors play a significant role in Hg accumulation (Pérez-Rodríguez *et al.*, 2018).

On the other hand, Rapa Nui has no industrial development; however, the human population and number of tourists visiting the island have increased considerably in recent decades (Vera *et al.*, 2022). The island does not possess any wastewater treatment plants; many houses have cesspools, a lower proportion use septic tanks as well as some hotels and restaurants, but without any further treatment (Zapata-Hernández *et al.*, 2022). Both rainwater, gathered through channels and collectors, and submarine groundwater discharge could represent a potential route of land-derived material inputs (e.g. sediments, sewage effluents and other pollutants) into coastal ecosystems (Zapata-Hernández *et al.*, 2022). Thus, the elevated Hg concentrations present in Rapa Nui turtles could be associated with local sources of pollution, both natural and anthropogenic. Likewise, long-range transport and accumulation of mercury within the same hemisphere, influenced by the oceanographic features of the Rapa Nui region, cannot be excluded (Schneider *et al.*, 2023).

### Hand-feeding as a source of mercury contamination in turtles of Rapa Nui

Hand-feeding turtles by fishermen with top predator fish (e.g. tuna or swordfish) is a frequent practice in Rapa Nui, particularly in sheltered bays and ports (Álvarez-Varas *et al.*, 2015, 2022). This practice has also been reported in other regions where fishermen provision foraging turtles with fish offal discarded from their boats, such as in Barbados, West Indies (Stewart *et al.*, 2016) and the Canary Islands (Monzón-Argüello *et al.*, 2018). Similarly, stable isotope studies have suggested a carnivorous diet for green turtles in the epipelagic zone associated with fishery discards (Turner Tomaszewicz *et al.*, 2018). Although little is known about the potential impacts of supplemental feeding on sea turtles, recent studies have shown that provisioned turtles exhibit not only behavioural and growth alterations but also changes in blood biochemical markers related to health status and increased pollutant loads (Lazar *et al.*, 2011; Bezerra *et al.*, 2014; Stewart *et al.*, 2016). Additionally, supplemental feeding increases turtle tameness, which heightens their vulnerability to unintended interactions with detrimental human activities, such as bait poaching and watercraft strike (Horrocks *et al.*, 2007; Monzón-Argüello *et al.*, 2018).

This local practice in Rapa Nui may also be contributing to elevated Hg levels in the turtles. Monzón-Argüello *et al.* (2018) studied green turtles from the Canary Islands and found that an increase in prey consumption was associated with abnormal levels of cholesterol, triglycerides and BUN, as well as elevated concentrations of both organic and inorganic pollutants, such as cadmium and lead. Similarly, based on contaminant and stable isotope analyses, Monzón-Argüello *et al.* (2018) suggested that benthic feeding or herbivory resulted in lower concentrations of organochlorine pesticides compared to the consumption of pelagic fish and cephalopods. Furthermore, cadmium concentrations were found to increase with trophic level. While this study highlights the potential impact of supplemental feeding with high-trophic-level fish on pollutant accumulation in green turtles, further research is needed to clarify the relationship between diet and contaminant concentrations in Rapa Nui turtles. These findings are also critical from a public health perspective, given the high levels of fish consumption within the local community.

### The impact of mercury contamination on the health of Rapa Nui turtles

Toxic effects of Hg have been linked to renal and hepatic damage, as well as reported neurotoxic, genotoxic and immunotoxic effects in marine vertebrates (Perrault *et al.*, 2017a; Kershaw and Hall, 2019). Particularly in sea turtles, studies have demonstrated that increased concentrations of mercury in nesting females may have detrimental effects on reproductive success (Perrault *et al.*, 2013). Investigations *ex vivo* and *in vitro* have confirmed a decrease of lymphocytes and suppression of proliferative responses for B cells associated with mercury exposure, suggesting negative impacts of this element on sea turtle immune function (Day *et al.*, 2007). Likewise, Perrault *et al.* (2017b) suggested a possible association with mercury and increased tumour growth in green turtles afflicted with fibropapillomatosis. Although we did not find a direct relationship between Hg and immunosuppressive biomarkers in Rapa Nui turtles, the presence of lymphopenia, toxic heterophils, hypoproteinaemia (low globulin and TP levels), elevated CPK and BUN levels, and a correlation between ALP and lymphocytes suggests altered immune function in these individuals (Aguirre *et al.*, 1995; Work *et al.*, 2001; Day *et al.*, 2007; Perrault *et al.*, 2017a, 2021; McNally and Innis, 2020).

Adams *et al.* (2010) used quantifiable pathological and biochemical indicators to investigate Hg-associated differences in marine fish from a highly contaminated site and a reference area in Florida, USA. Liver histology revealed pyknosis/necrosis, interstitial inflammation, and bile duct hyperplasia exclusively in fish from the contaminated site. Similarly, interstitial inflammation, glomerular dilatation and thickening, and tubular degeneration and necrosis in renal tissue were more frequently observed at the contaminated site, demonstrating a significant impact of Hg on both organs (Adams *et al.*, 2010). In loggerhead turtles, a correlation has been reported between BUN and Hg concentrations, which has been interpreted as dietary exposure to the metal in actively foraging individuals (Perrault *et al.*, 2017a). Although we did not find a direct relationship between BUN and Hg, our results showed a positive correlation between urea and Hg, along with elevated BUN levels in the turtles from Rapa Nui. Urea is produced in some aquatic reptiles as a primary nitrogenous waste product of protein catabolism (Allender and Latney, 2016). Elevated urea levels are typically associated with kidney failure, dehydration, a high-protein diet or excessive protein breakdown (Delgado *et al.*, 2011). Given the low TP levels in our study, it is likely that the observed relationship between urea and Hg, along with the increased BUN, is not diet-related but rather indicative of renal dysfunction. Similarly, a previous study in loggerhead turtles reported increased CPK levels linked to Hg exposure, likely associated with muscle and/or renal damage (Day *et al.*, 2007). The correlation between Hg and urea, along with the elevated CPK and BUN levels suggesting renal damage, may also be supported by hypocalcaemia, low creatinine levels, and the correlations between creatinine and calcium, and Hg and calcium, observed in the feeding aggregation of Rapa Nui (Delgado *et al.*, 2011; Allender and Latney, 2016).

In reptiles, AST is an enzyme primarily found in the kidneys and, to a lesser extent, in the liver. It should be present in the bloodstream at low concentrations (Tristan, 2008; Cannavacciuolo, 2013). Elevated AST levels in circulation indicate renal damage (Pagano *et al.*, 2019). Additionally, in many reptiles, increased AST levels coupled with decreased cholesterol may suggest hepatocellular injury (Allender and Latney, 2016). In marine turtles, elevated Hg concentrations are thought to lead to lipid peroxidation, reducing cholesterol levels (Perrault *et al.*, 2017a). Thus, the positive correlation between AST and Hg, along with low cholesterol levels observed in this study, may indicate liver dysfunction linked to Hg exposure in Rapa Nui turtles. However, these results should be interpreted with caution given the small sample size of this study.

Further research with a larger sample size will enhance our understanding of Hg impacts on Rapa Nui turtles and inform strategies to mitigate its effects on this isolated population in the southeastern Pacific. Evaluating Hg levels in key food sources, while accounting for variations in morphotype and life stage, is crucial, as diet is the primary route of Hg exposure. Additionally, monitoring Hg concentrations in sediments, water columns, and particularly in leftover fish in Hanga Roa, will provide a more comprehensive view of environmental contamination and enable prompt mitigation actions. Satellite tracking of turtle movements will also help identify or rule out pollution sources and guide management strategies. This is critical for public health on this tourist island, where humans and turtles interact year-round. All of this information will be essential for the implementation of the Rapa Nui Multiple Uses Marine Protected Area, where *C. mydas* is a conservation priority.

## Data Availability

The data underlying this article will be shared on reasonable request to the corresponding author.
